# Quantitative estimation of genetic risk for atypical scrapie in French sheep and potential consequences of the current breeding programme for resistance to scrapie on the risk of atypical scrapie

**DOI:** 10.1186/1297-9686-42-14

**Published:** 2010-05-18

**Authors:** Alexandre Fediaevsky, Didier Calavas, Patrick Gasqui, Katayoun Moazami-Goudarzi, Pascal Laurent, Jean-Noël Arsac, Christian Ducrot, Carole Moreno

**Affiliations:** 1 INRA, Centre de Clermont-Theix, 63122 Saint Genès Champanelle, France; 2 AFSSA-Lyon, 31 Avenue Tony Garnier, 69364 Lyon Cedex 07, France; 3 INRA GABI, UMR1313, domaine de Vilvert, 78252 Jouy-en-Josas, France; 4 INRA, SAGA, UR631, BP52627, 31326 Castanet-Tolosan, France

## Abstract

**Background:**

Since 2002, active surveillance programmes have detected numerous atypical scrapie (AS) and classical scrapie cases (CS) in French sheep with almost all the PrP genotypes. The aim of this study was 1) to quantify the genetic risk of AS in French sheep and to compare it with the risk of CS, 2) to quantify the risk of AS associated with the increase of the ARR allele frequency as a result of the current genetic breeding programme against CS.

**Methods:**

We obtained genotypes at codons 136, 141, 154 and 171 of the PRNP gene for representative samples of 248 AS and 245 CS cases. We used a random sample of 3,317 scrapie negative animals genotyped at codons 136, 154 and 171 and we made inferences on the position 141 by multiple imputations, using external data. To estimate the risk associated with PrP genotypes, we fitted multivariate logistic regression models and we estimated the prevalence of AS for the different genotypes. Then, we used the risk of AS estimated for the ALRR-ALRR genotype to analyse the risk of detecting an AS case in a flock homogenous for this genotype.

**Results:**

Genotypes most at risk for AS were those including an AFRQ or ALHQ allele while genotypes including a VLRQ allele were less commonly associated with AS. Compared to ALRQ-ALRQ, the ALRR-ALRR genotype was significantly at risk for AS and was very significantly protective for CS. The prevalence of AS among ALRR-ALRR animals was 0.6‰ and was not different from the prevalence in the general population.

**Conclusion:**

In conclusion, further selection of ALRR-ALRR animals will not result in an overall increase of AS prevalence in the French sheep population although this genotype is clearly susceptible to AS. However the probability of detecting AS cases in flocks participating in genetic breeding programme against CS should be considered.

## Background

Bovine spongiform encephalopathy (BSE) is a zoonotic transmissible spongiform encephalopathy (TSE), which may have spread to sheep and goat populations. This situation has prompted European countries to organise control and surveillance of TSEs in small ruminants in which BSE cannot be clinically distinguished from scrapie.

In sheep, genetic susceptibility to classical scrapie (CS) is supported by polymorphic variations at codons 136, 154 and 171 of the PRNP gene, which encodes the normal cellular protein prion (PrP^C^) [1]. The main alleles defined by these three codons can be classified by increasing risk as follows ARR < AHQ < ARQ ≈ ARH < VRQ [2]. This knowledge has made it possible to implement eradication programmes throughout Europe based on positive selection of the most resistant allele (ARR) and negative selection of the most susceptible one (VRQ) [2,3].

Recently, an atypical form of scrapie (AS) has been detected and has challenged such programmes since animals genetically resistant to CS are affected [4-7]. However, although it is generally accepted that CS is an infectious and contagious disease [8], the contagiousness of AS is questioned. Indeed, the marker specific for AS disease is not detected outside the central nervous system [5,9-14], even in cases where AS has been experimentally transmitted to transgenic mice [15] and sheep [16].

Furthermore, most AS cases have been observed in animals with genotypes showing low susceptibility to CS. Several studies have shown that AS susceptibility is highly associated with PrP codons 141 (L/F) and 154 (R/H) [17,18]. It is important to note that AS cases have been detected in ALRR carriers but rarely in VLRQ carriers.

A descriptive study has reported that the estimation of AS prevalence in Europe is similar, whatever the country or tested population and amounts to about 6 cases per 10,000 tests. Conversely, CS prevalence appears to be heterogeneous both between countries and, within countries, between fallen stock and healthy slaughter [11]. This could hide a relatively high prevalence of AS in the most susceptible genotypes. In addition, estimation of the prevalence of AS in ALRR-ALRR animals is strategically important to assess the impact of current breeding programmes on the AS prevalence in the general population. In France, breeders of flocks participating in breeding programmes for CS resistance are encouraged to join in a voluntary scrapie certification scheme, for which it is also compulsory to send animals to breed selection centres (Internal circular from French Ministry of Agriculture, DGAL/SDSPA/N2006-8093). This certification scheme implies testing all fallen stock over 18 months, which increases the risk of detecting AS cases in these flocks compared to general flocks.

We have carried out a case control study by using data from the TSEs surveillance programme to estimate the risk of AS associated with the PrP genotypes in the French sheep population and to compare it with the risk of CS. In addition, we have used our dataset to estimate the prevalence of AS per genotype and the risk for a fully CS resistant flock (100% ALRR-ALRR) to be detected positive for AS under certain assumptions.

## Methods

### **Selection of cases**

Cases were recruited among the animals tested for TSEs in France from 1^st ^January 2002 to 31^st ^December 2007. Each year since 2002, a number of adult (over 18 months old) sheep from unknown TSE status flocks was randomly selected for TSE detection at abattoirs (healthy slaughter) or at rendering plants (fallen stock). Details concerning the French active surveillance programme are described in Cazeau [19]. Between 2002 and 2007, 859,157 samples were analysed within this programme, among which 532,500 were tested with the recommended tests for AS detection [20,21].

All positive samples were confirmed and typed by the National reference laboratory for animal TSEs of AFSSA Lyon using EU approved reference methods [22]. For all positive cases the presence of BSE was dismissed by discriminatory tests; the cases detected with the tests recommended for AS were typed in order to classify them as AS or CS; other positive cases were assumed to be CS. During that period, 404 cases of AS and 395 cases of CS were confirmed. Among these cases, DNA was available or could be extracted from the central nervous system samples kept at AFSSA Lyon for respectively 248 AS and 245 CS cases.

### Genotyping of CS and AS cases

During that period, the PrP genotype of all cases was routinely determined, when suitable samples were available, at codons 136, 154 and 171 with a technique that could not differentiate ARH and ARQ alleles. Further analyses were conducted to specify alleles at position 141 and 171 by the Taq man method [23,24]. It is generally agreed that this method provides accurate, reproducible and reliable results. For example, in human large-scale association studies, the error rate is estimated to be less than 0.3% [25] or even 0.05% [26]. These analyses were conducted by the National reference laboratory for PRNP genotyping (Labogena, Jouy-en-Josas, France) which is certified for this test by the French standard COFRAC (NF EN ISO/CEI *17025)*. PrP polymorphisms at codons 136, 141, 154 and 171 of the 248 AS cases were analysed after direct sequencing of the coding region of the PRP gene encompassing codons 92-282. These analyses were conducted by INRA (Jouy-en-Josas, France). For more details concerning primers and sequencing see [27].

### Selection of controls

Controls were recruited among animals that were tested for the presence of TSEs with a negative result. Controls for AS were animals tested with one of the tests recommended for the detection of AS and controls for CS were animals tested with any rapid test.

### Genotyping of controls

As a legal requirement [3], during the TSE active surveillance programme 2002-2007, a subset of 3,347 animals was randomly selected for PrP genotyping. We used this sample as genotype controls. The genotyping of this control dataset was conducted by Labogena at codons 136, 154 and 171 with the limits specified before (missing information for codons 141 and 171). No significant difference of PrP frequencies at codons 136, 154 and 171 was observed between the present control dataset and estimates from an external data set of the general French ovine population [27]. In addition, distributions in both datasets were in Hardy-Weinberg equilibrium. For these two reasons, we inferred F141 and H171 information from the external data set to our control data set. In order to take into account a possible uncertainty on the estimation of F141 and H171 in the external dataset, we considered that the estimated values for F141 and H171 were random variables with beta-multinomial distributions with means and variances equalling those estimated from the external dataset. Finally, we took into account that uncertainty in the inference process by bootstrapping 1,000 times the control dataset.

### Demographic data

In order to adjust for potential confounding variables, we collected information on the stream of surveillance (healthy slaughter/fallen stock) and the dentition of the animals (2-4 definitive incisors (DI)/5-7 DI/8 DI) which is a poor proxy for age [28] but was the only information available. This led to exclude some animals for which information was missing (two cases of CS and nine controls).

### Multivariate analysis

For each type of scrapie, we fitted multivariate logistic regression models to assess the risk associated to genotypes with ALRQ-ALRQ as the baseline category, because this genotype is assumed to be the ancestral genotype. We adjusted the models on the surveillance stream with healthy slaughter as the baseline category, and on dentition, with the class corresponding to the youngest animals as the baseline category. The model outputs were odds ratio (OR) which can be interpreted as an approximation of the relative risk in the context of rare diseases. We could not estimate OR associated with the genotypes in which no case was detected. The value of the OR and their 95% confidence intervals for the 1,000 datasets were computed using the Rubin method [29].

### Estimation of prevalence

We estimated the prevalence of AS for each genotype. Because the proportion of animals genotyped was different for cases and controls, we defined it as the national prevalence (404/532,500 for AS) times the proportion of the genotype among cases, divided by the frequency of the genotype in the general population. Using a Bayesian approach, we considered that each of these proportions followed a beta distribution with mean and variance estimated by the observations. We derived the median and the 95% credibility interval of prevalences based on 2,000 iterations obtained after convergence.

### Estimation of the probability of case detection at flock level

We have considered the risk for a fully ALRR-ALRR flock to be detected positive for AS. We assumed that in any flock the individual risk of AS depended only on the genotype, the age and the surveillance stream. Considering that the animals tested from a given flock had the same distribution of age and surveillance stream as the general population, the average individual risk for an ALRR-ALRR animal could be estimated by the prevalence previously estimated for this genotype. Since the occurrence of AS in a flock was defined as an independent event, the number of cases in a flock, X, followed a binomial distribution. The probability for at least one animal being AS positive among *n *animals tested was:(1)

Using the previous methods to estimate the prevalence per genotype, we derived the median of the probability and its 95% credibility interval for n varying from 1 to 1,000, based on 2,000 iterations obtained after convergence.

All the statistical analyses were done with R for Windows [30] and Winbugs [31].

## Results

After exclusion of missing information on genotypes and dentition, 248 AS cases, 245 CS cases and 3,317 controls were used to estimate OR and prevalence (Table 1). Cases of CS and/or AS were detected in all genotypes.

**Table 1:** Risk of AS and CS according to genotypes

		AS			CS	
Genotype	No controls*	No cases	**OR**^**†**^**(CI 95%)**^**‡**^	**Prevalence ‰ (ci 95%)**^**§**^	No cases	OR (CI 95%)
ALRR-ALRR	772	45	12.7 (2.9-55.1)**	0.60 (0.45 - 0.78)	0	NA^††^
ALRR-ALHQ	70	24	75.2 (16.6-340.6) **	3.56 (2.21 - 5.59)	0	NA^††^
ALRR-AFRQ	147.4	54	82.9 (13.6-506.5)**	3.77 (2.80 - 5.02)	0	NA^††^
ALRR-ALRQ	1003.4	10	2.2 (0.5-10.0)	0.11 (0.06 - 0.19)	2	0.0 (0.0-0.1)^†^
ALRR-ALRH	126.1	3	5.3 (0.6-46.4)	0.30 (0.09 - 0.72)	0	NA^††^
ALRR-VLRQ	188	0	NA^††^	0 (0.0 - 0.19)	16	0.5 (0.2-1.0)^‡‡^
ALHQ-ALHQ	3	6	454.8 (61.2-3381.4)**	19.47 (5.61 - 75.07)	1	1.4 (0.1-15.7)
AFRQ-ALHQ	9.8	11	253.5 (29.0-2213.8)**	11.45 (5.16 - 27.48)	0	NA^††^
ALHQ-ALRQ	65.9	13	41.6 (9.0-192.2)**	2.11 (1.10 - 3.51)	1	0.1 (0.0-0.8)^‡‡^
ALHQ-ALRH	8.4	2	54.1 (4.2-689.3) ^‡‡^	3.03 (0.58 - 10.01)	0	NA^††^
ALHQ-VLRQ	4	1	51.1 (3.7-709.0)^‡‡^	3.55 (0.44 - 18.14)	2	2.0 (0.3-12.1)
AFRQ-AFRQ	11.6	28	724.3 (49.4-10625.1)**	23.56 (12.65 - 47.08)	2	1.5 (0.1-22.1)
AFRQ-ALRQ	126.4	43	76.9 (13.5-438.7)^†^	3.50 (2.44 - 4.74)	20	1.0 (0.3-3.2)
ALRQ-ALRQ	445.5	2	Baseline category	0.06 (0.01 - 0.17)	69	Baseline
ALRQ-ALRH	106.7	0	NA^††^	0 (0 - 0.37)	8	0.5 (0.1-1.8)
AFRQ-ALRH	16.5	1	15.7 (0.7-335.4)	1.01 (0.15 - 3.61)	2	1.0 (0.1-12.2)
ALRH-ALRH	9.7	1	33.8 (1.1-1008.5) ^‡‡^	1.73 (0.29 - 6.61)	1	1.0 (0.0-24.6)
AFRQ-VLRQ	21.5	3	31.7 (3.4-293.4)	1.71 (0.50 - 4.64)	6	1.7 (0.3-8.2)
ALRQ-VLRQ	147.1	0	NA^††^	0 (0.00 - 0.26)	85	3.6 (2.3-5.6)**
ALRH-VLRQ	18.4	1	12.7 (0.8-212.6)	0.91 (0.13 - 3.27)	9	3.2 (0.6-17.1)
VLRQ-VLRQ	16	0	NA^††^	0.45 (0.02 - 2.48)	21	7.8 (3.3-18.1)^†^
General population				0.76 (0.69 - 0.83)		
Healthy slaughter			Baseline category			Baseline
Fallen stock			1.0 (0.7-1.3)			6.44 (4.43-9.37)**
2-4 DI			Baseline category			Baseline
5-7 DI^§§^			1.6 (0.4-6.5)			1.04 (0.43-2.49)
8 DI***			3.0 (0.8-11.4)			1.07 (0.46-2.50)

* Mean value for the 1,000 bootstraps

^† ^Odds Ratio

^‡ ^95% confidence interval

^§ ^95% credibility interval

** p-value < 0.001

^†† ^Not available: no individual with that genotype

^‡‡ ^p-value ≤ 0.05

^§§ ^5-7 definitive incisors: 2- to 4-year old animals

*** 8 definitive incisors: animals of 4 years old and more

There were no AS cases among the ALRR-VLRQ, ALRQ-ALRH, ALRQ-VLRQ, and VLRQ-VLRQ genotypes. The ALHQ-ALHQ, AFRQ-ALHQ and AFRQ-AFRQ genotypes were associated with the highest risks of AS compared to ALRQ-ALRQ. Within CS cases, the VLRQ-VLRQ animals presented the highest risk compared to ALRQ-ALRQ. ALRR carriers and to a lesser extent ALHQ carriers were the most resistant to CS.

The AS prevalence estimates for the most susceptible genotypes (AFRQ/ALHQ, ALHQ/ALHQ and AFRQ/AFRQ) were respectively 11, 25 and 31 times higher than the prevalence for the general population (Table 1). The AS prevalence estimate for ALRR-ALRR animals was not significantly different from that in the general population.

The animals tested at fallen stock were significantly more at risk for CS but not for AS. Adjustment on the dentition of the animals improved the fit of the model for AS (log-likelihood ratio test of models with and without dentition: p-value <0.003), the oldest animals were more at risk but OR was not significantly different from 1 at 5%. However, this could be due to a lack of power since comparison with the intermediate category showed that the OR associated with the oldest category was significantly different from 1. For CS there was no observed effect of age.

**Figure 1:**
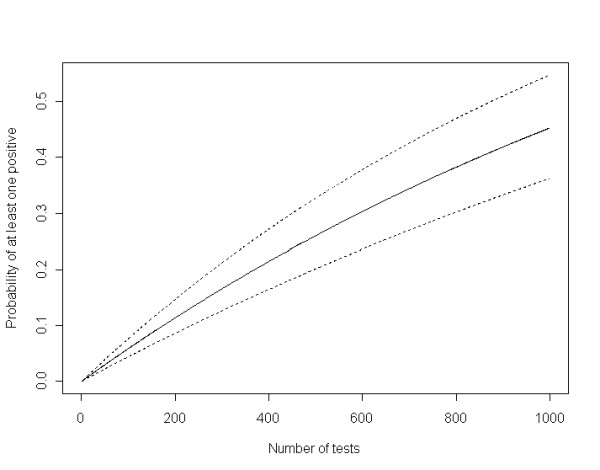
**Probability for a 100% ALRR-ALRR flock to have at least one AS case detected according to the number of animals tested**. Plain line: median of probability, dashed lines: 95% credibility interval of probabilty

The probability for a 100% ALRR-ALRR flock to be detected as positive for AS was not negligible when 100 animals were tested (Figure 1). For instance, a flock with a constant size of 400 ALRR-ALRR adult ewes and an adult mortality rate of 5% would have 200 animals tested over a 10 year-period of time. Under these conditions, the cumulative probability over 10 years to detect at least one AS case out of 200 animals tested for TSEs would be 10%.

## Discussion

This study was aimed at estimating the genetic risk for AS in French sheep and potential consequences of the current breeding programme for the ARR allele on the risk of atypical scrapie. The analysis was conducted on a sheep population submitted to TSE active surveillance and we have considered the ancestral genotype ALRQ-ALRQ as the baseline reference. Our results were congruent with those from other studies either regarding ranking AS susceptibility [12,17,27,32] or AS genotype specific prevalence [33]. We detected a significant risk of AS for sheep carrying the ALRR-ALRR genotype.

The risk and prevalence of AS for AFRQ-AFRQ, AFRQ-ALHQ and ALHQ-ALHQ genotypes were the highest. Besides, AFRQ-AFRQ and ALHQ-ALHQ homozygotes had a much higher risk than AFRQ-ALRQ and ALHQ-ALRQ heterozygotes which is not indicative of a dominant effect.

The effect of the VLRQ allele towards AS was not obvious. No or very few cases of AS have been detected among VLRQ carriers, depending on the other allele, but VLRQ carriers are also relatively rare. So the apparent protective effect cannot be properly confirmed and such an effect would need to be assessed through a larger study, at the European scale for example.

Our estimations were based on a control dataset and a case dataset both originating from the population submitted to TSE active surveillance which is representative of adult animals collected at rendering plants or slaughtered for human consumption. Both datasets were randomly selected and we did not identify any selection bias linked with the exclusion of missing data within case and control datasets.

A strong assumption to establish such an estimation is that population and risk are homogenously distributed. In France, this assumption appears to be verified for AS but not for CS for which an important spatial heterogeneity is observed [14,34]. This could be due to differential exposure to CS infectious agent and/or different strains of CS having different behaviours in terms of genetic susceptibility [1,35]. For this reason, we have decided not to present genotype specific prevalence for CS. Matching cases and controls could have been an option. However that was not possible in our design, which used the subjects retrospectively selected in compulsory TSE active surveillance.

Our knowledge of the general French sheep population genotype structure was based on an extensive survey for the codons 136, 154 and 171 but it was based on an unbiased but much smaller sample for the codon 141. We took into account that difference of accuracy and the resulting uncertainty by bootstrapping the control dataset, which eventually increased the robustness of our results.

The maintenance of the polymorphism of the PRNP gene raises question. This could be explained by an absence of selection due to the late onset of clinical signs in the life of farm animals. Alternatively, it could be maintained by a balancing selection process [36,37]. The weak associations between PrP alleles and the production performances or health traits found so far [38] would hardly contribute to this selection. However, recent results have shown the selective advantages of susceptible PrP alleles on survival traits or on physical breed characteristics [39,40]. Another explanation may be found in heterozygous advantage and/or frequency dependency. The frequency dependent selection is supported by the differences of genetic susceptibility associated to PrP alleles towards the numerous strains of scrapie that prevail in the different subpopulations. This diversity includes AS and the many strains of CS [41]. There is also a possible advantage of heterozygous compared to homozygous individuals, specific to AS and suggested by the lower risk for AFRQ-ALHQ animals compared to ALHQ or AFRQ homozygous animals found in our study and in Great Britain [42].

The susceptibility to AS conferred by the ALRR allele questions the long term consequences of the breeding programme for CS resistance on AS prevalence in the sheep population and particularly in the flocks deeply engaged in this programme. The AS prevalence estimated for the ALRR-ALRR subpopulation (0.60‰, CI95%: 0.45-0.78) was not significantly different from the current average national prevalence (0.76‰, CI95%: 0.69-0.83). Therefore in standing conditions and assuming AS occurrence is not dependent on infectious exposure, increasing frequency of the ALRR allele in the general population should not result in an increase of the global AS prevalence in the sheep population.

Despite the low prevalence of AS in ALRR-ALRR animals, the probability of detecting a positive case is not negligible when the number of animals tested increases as shown by Figure 1. Farms fully engaged in ALRR-ALRR selection tend to test more animals due to their participation in the scrapie certification scheme, thus the probability to detect AS in their flock should be considered. The consequence is that they face a risk of being placed under inadequate control measures.

The assumption of a common risk of AS in farms is supported by the homogenous distribution of the disease and similarities of prevalence per genotype in France and in Europe [11,19,33]. This is partly challenged by the existence of some potential risk factors, such as mineral feeding [9,13]. However, in a recent case-control study, the farm level risk factors were much less important than the genetic factors [13]. Actually a number of studies argue for the hypothesis that AS could develop in the absence of exposure to an infectious agent [5,9-13,43], even if a doubt persists given the limited knowledge on the physiopathology of AS. Genotype appears as a very strong component for risk of AS and possibly age too. To have a better understanding of their respective impact on the development of the disease it would be useful to study the age-genotype interactions. This would especially help to assess age-specific penetrance of the different genotypes and to have an insight on the survival of animals with very susceptible genotypes. Unfortunately to carry out such assessments, more detailed data on age of cases and control populations and larger populations are required. Careful monitoring of AS positive flocks as planned by new European approaches on the control of AS could help address this issue in the future [3].

## Conclusion

Finally, AS occurrence appears to be heavily dependent on the PrP genotype, possibly modulated by some environmental risk factors which are to be further explored. Even if the open reading frame of the PRNP gene has a strong influence on the occurrence of AS it might not be the unique genetic factor of susceptibility. Other regions of the PRNP or other genes could be involved either independently or in synergy and this hypothesis is also worth to be investigated.

## Competing interests

The authors declare that they have no competing interests.

## Authors' contributions

AF, PG, CD, DC and CM conceived the study, participated in its design and coordination. AF and CM performed the strategy of the selection of animals and carried out the statistical analysis. JNA performed the scrapie status confirmation and the strain determination. PL and KMG performed the polymorphism genotyping work by sequencing. AF, PG, CD, DC, CM, JNA and KMG helped to draft the manuscript. All authors read and approved the final manuscript.
